# Changes in surgical therapies for rectal cancer over the past 100 years: A review

**DOI:** 10.1002/ags3.12342

**Published:** 2020-05-10

**Authors:** Yuji Toiyama, Masato Kusunoki

**Affiliations:** ^1^ Department of Gastrointestinal and Pediatric Surgery, Division of Reparative Medicine Institute of Life Sciences, Mie University Graduate School of Medicine Tsu Japan

**Keywords:** radiation therapy, rectal cancer, surgery

## Abstract

Advances in surgical and adjuvant therapies have resulted in a dramatic improvement in outcomes of rectal cancer in terms of both oncology and functional preservation. Surgery plays a central role in therapy as it is the only means of achieving a complete cure. These surgical advancements result from extensive pioneering research in the fields of anatomy and physiology. Much history lies behind the recent surgical breakthroughs of total mesorectal excision (TME) and intersphincteric resection (ISR). This article outlines the changes that have taken place in surgical therapies for rectal cancer over more than a century based on clinical trials performed to provide scientific evidence for these therapies.

## Graphical abstract

Figure 1: Representative approach to rectal surgery. Figure 2: Sphincter‐saving restorative procedures.

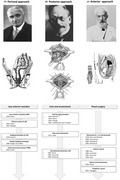

## 1 INTRODUCTION

Rectal cancer surgery is a surgical system that has developed as both an art and a science since first being introduced in the early 19th century. This was a time of insufficient anesthesia and poor infection control. At this time, the main focus of rectal cancer surgery was the performance of the resection. To this end, surgical approaches through the rectum were devised with the goal of improving resection rates. Typical approaches were the perineal, posterior, and anterior approaches, which were sometimes combined, and were intended to achieve complete resection. Thereafter, a desire to preserve anal function led to the development of pull‐through procedures, a style of abdominal‐transanal resection and anastomosis. These ultimately led to pouch surgeries and ISR to preserve the natural anus. In parallel, surgical styles have developed from an oncological point of view, and laparoscopic and robot‐assisted surgeries have appeared as minimally invasive procedures aimed at achieving early postoperative recovery. In the following sections, we will explore, in detail, surgical approaches for rectal cancer.

## 2 SURGICAL APPROACHES FOR RECTAL CANCER

### 2.1 Perineal approach

In 1826, Lisfranc successfully performed the world's first perineal rectal amputation for lower rectal cancer. However, operations at that time were performed via a perineal approach as en bloc resections of the perineum, including the rectum and anus. Without a stoma, there was uncontrollable excretion through the perineum.^1^

In 1926, Lockhart‐Mummery proposed a technique in which a permanent colostomy was constructed in advance and, after resecting the rectal cancer including the perineum, the sigmoid colon stump was closed and retained in the abdominal cavity. The mortality rate with this procedure was 8.5%, which was considered good. In 1932, Gabriel et al reported resection, mortality, and 5‐year survival rates of 50%, 11.6%, and 40%, respectively, with this procedure.^2^

**Figure 1:**
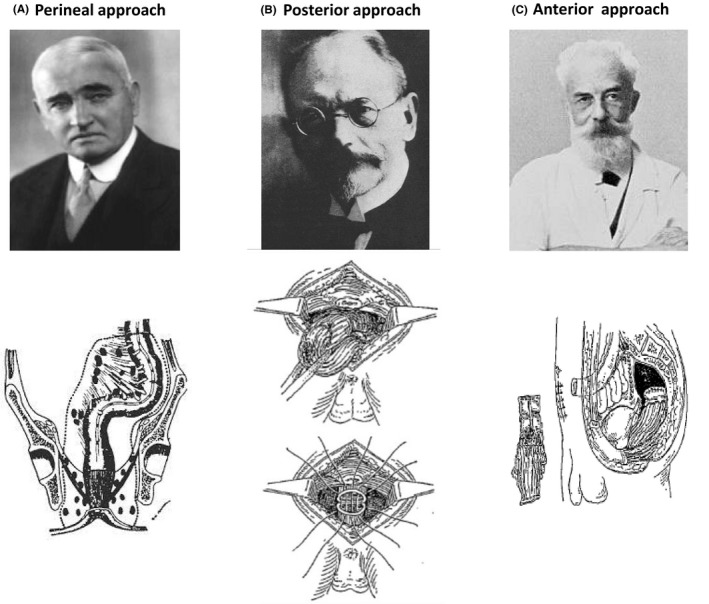
Representative approach to rectal surgery. A, An original procedure for rectal cancer reported by Dr Ernest Miles in 1908, which is a combination of the perineal and anterior approach. Cited from the references: 1. Campos FG. The life and legacy of William Ernest Miles (1869‐1947): a tribute to an admirable surgeon. Rev Assoc Med Bras (1992) 59:181‐185, 2013. 2. Campos FG, Habr‐Gama A, Nahas SC, et al: Abdominoperineal excision: evolution of a centenary operation. Dis Colon Rectum 55:844‐853, 2012. B, An original procedure for rectal cancer reported by Dr Paul Kraske in 1885 as one of the posterior approaches. Cited from the reference: 1. Classic articles in colonic and rectal surgery. Paul Kraske 1851‐1930. Extirpation of high carcinomas of the large bowel. Dis Colon Rectum 27:499‐503, 1984. (C) An original procedure for rectal cancer reported by Dr Henri Hartmann in 1921 as one of the anterior approaches. Cited from the reference: 1. Zbar AP. Henri Albert Hartmann (1860‐1952): colorectal master Tech Coloproctol.12:175‐179, 2008

In search of more complete cures, rectal cancer surgery, which originated with the perineal approach, was then combined with abdominal cavity approaches. In 1908, Miles reported an abdominal‐perineal style of rectal amputation as a radical procedure for rectal cancer (Figure 1A).^3^ Miles' pathological and anatomical analyses revealed that most recurrences were identified in the pelvic peritoneum, the pelvic mesocolon, and the lymph nodes around the bifurcation of the left common iliac artery. He named this the "zone of upward spread" and proposed en bloc resections of this region where micrometastases were likely to spread. This was designed to prevent the recurrence of rectal cancer.^3^ Although this was a groundbreaking oncological concept, the procedure had significant safety problems, with 22 of 66 patients (33.3%) dying during or soon after surgery.^2^ Later advancements in anesthesia and perioperative management made it easier to mobilize the rectum from the abdominal cavity approach, and the appearance of the lithotomy position and the development of lithotomy stirrups eliminated intraoperative position changes; these factors all made the Miles operation easier to perform. In 1939, Lloyd‐Davies shortened the operation time and increased safety by developing the Lithotomy‐Trendelenburg position, which made it possible to perform abdominal and perineal manipulations simultaneously.^4^ Against this background, the original oncological concept of the Miles operation resulted in good survival rates and was widely accepted worldwide. It has long been established as the standard procedure for rectal cancer surgery.most recurrences were identified in the pelvic peritoneum, the pelvic mesocolon, and the lymph nodes around the bifurcation of the left common iliac artery.

### 2.2 Posterior approach

Because the perineal approach of Lisfranc et al involved problems with the field of vision for manipulations in the upper rectum, Kocher et al in 1876 reported a technique for performing rectal resection and primary anastomosis by securing a visual field through resection of the coccyx and part of the sacrum.^5^ This procedure developed into the Kraske procedure, which is a famous transsacral approach for rectal cancer (Figure 1B). Based on postmortem studies, Kraske proposed that the upper rectum could easily be mobilized by incising the gluteus maximus and levator ani from the left side of the sacrum, and in 1886 reported a procedure using this method. Good sphincter functions were observed with this procedure, but there were complications involving pelvic fistulas.^5^ It has been pointed out that sphincter damage and other problems may occur during detachment or suturing with the Kraske surgery, and that one‐stage anastomosis can be visually difficult with only the sacral approach.^2^ To resolve these issues, Localio et al reported the usefulness of a combined sacral and abdominal cavity approach.^6^ In 1969, they reported an abdominal‐sacral approach in the lateral recumbent position, in which full mobilization of the rectum allowed for safe preservation of the sphincter and one‐stage anastomosis.^6^ They reported the postoperative outcomes of 427 rectal cancer patients who underwent the anterior approach described below, the Miles operation, or an abdominal‐sacral approach. The abdominal‐sacral approach was used in 100 patients, among whom the recurrence and mortality rates were not inferior to that with the other procedures. However, the abdominal‐sacral approach had a high rate (12%) of pelvic fistulas, peritonitis, and other postoperative complications. Therefore, they advised also performing a colostomy when the abdominal‐sacral approach was used.^2^

As an alternative posterior approach, Mason, in 1970, proposed a transsphincter approach.^7^ Known as a posterior approach for local resection, this procedure was initially developed from the abdominal‐sacral approach. This technique involves temporarily separating the internal and external sphincters and the levator ani, performing the rectal resection under direct vision, then repairing the separated sphincter group after anastomosis. However, due to the high local recurrence rate with this procedure, confirmation of the surgical margin by intraoperative rapid pathological examination is recommended.^7^

### 2.3 Anterior approach

In 1921, Henri Hartmann reported what is now called Hartmann's operation, in which the upper rectum was resected from the abdominal cavity and a single‐barreled colostomy was created without anastomosis (Figure 1C).^5^ The original method was a two‐stage operation, in which a colostomy was first created, followed by resection of the rectum. This procedure resulted in a lower postoperative mortality rate than the Miles operation due to decreased blood loss.^5^ Because upper rectal cancer is known to have little downward lymphatic flow, Dixon of the Mayo Clinic reported an anterior resection procedure in which the rectal resection was performed from the abdominal cavity and anastomosis was performed in two layers.^8^ This procedure, known as the Mayo Clinic operation, became the standard procedure for rectal cancer originating from the upper rectum to the rectosigmoid junction. Morgan et al reported that the Mayo Clinic operation had similar outcomes to the Miles operation in terms of safety and completeness of cure.^9^ Rectal cancer surgery had entered the era of preservation of the natural anus. In 1978, Ravitch et al developed a gastrointestinal suturing device that made it possible to perform highly complex end‐to‐end anastomosis after rectal or esophageal resection, which became known as the stapling technique of lower anterior resection (LAR).^10^ In 1980, Knight et al reported the double stapling technique,^11^ which greatly contributed to the dissemination of anterior resection, and remains the standard technique today.

### 2.4 Transitioning to preserving anal function in rectal cancer

#### 2.4.1 Pull‐through procedure

**Figure 2:**
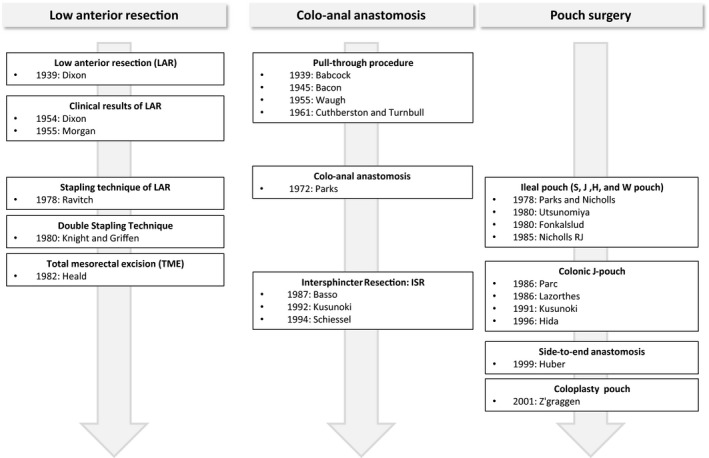
Sphincter‐saving restorative procedures

The pull‐through procedure involves resecting the lower rectum via a perineal approach, followed by coloanal anastomosis (Figure 2). Around 1940, Babcok and Bacon both reported pull‐through procedures, which were transanal‐abdominal procedures in which the rectum was resected while preserving the external anal sphincter, the mobilized colon was pulled about 50 cm outside the anus, and 2‐3 weeks later, the prolapsed intestine was removed.^12^, ^13^ In 1952, Black improved this procedure to allow for preservation of the internal and external sphincters.^2^ Turnbull and Cuthbertson used a technique in which, after rectal resection, the remaining rectal stump was everted and pulled out of the anus, and the colon was then pulled through this outside the abdominal cavity. In a second‐stage procedure, the excess colon was removed, anastomosis was completed outside the pelvis, the stump was turned back over, and the anastomosis placed inside the pelvis. This procedure was carried out for rectal cancer and Hirschsprung disease.^14^

In 1972, Parks reported a procedure in which the rectum was resected up to the level of the levator ani, mucosal resection of the upper anal canal was performed via an anal approach, and the pulled through colon was anastomosed transanally at the level of the dentate line.^15^ As a result of anatomical and physiological studies, Parks concluded that it was not always necessary to leave 6‐8 cm of residual rectal‐anal canal, and that it was more important to not damage the sphincter or pelvic floor muscles.^2^ The Parks procedure, which was a dramatic advancement over the conventional pull‐through procedure, did not gain popularity because of safety issues such as postoperative infection control, as well as its high degree of difficulty.

Thereafter, our predecessors, who attempted to preserve anal and defecation functions as much as possible, later developed two procedures—colonic pouch surgery and intersphincteric resection (ISR).

#### 2.4.2 Colonic pouch surgery

Defecation functions comprise rectal reservoir and anal sphincter functions. In order to maintain reservoir functions, Parc and Lazorthes developed the colonic J‐pouch in 1986. This was based on the total colectomy and ileal J‐pouch—anal anastomosis performed for ulcerative colitis and familial adenomatous polyposis.^16^, ^17^ However, while the ileal J‐pouch prevented fecal incontinence and increased defecation frequency, the colonic J‐pouch resulted in difficulty with defecation.

In 1991, Kusunoki et al first performed a randomized controlled trial (RCT) comparing colonic J‐pouch reconstruction to straight reconstruction for low rectal cancer, and found that postoperative defecation functions were significantly better with colonic J‐pouch reconstruction.^18^ Later, Hida et al^19^ and Lazorthes et al^20^ reported that the 5‐cm pouch was associated with better defecation functions, while overdistention and flattening along the long axis with the 10‐cm pouch made defecation difficult. Thus, reconstruction with a 5‐cm colonic J‐pouch is currently the standard.

As the optimal size of a colonic J‐pouch is small at approximately 5 cm, coloplasty pouches and side‐to‐end anastomoses were tried as alternative methods. In 2001, Z'graggen et al reported on coloplasty pouches for the first time, finding good defecation function 8 months after surgery in 41 cases.^21^ Mantyh et al found no difference in defecation or reservoir functions with coloplasty pouches compared to that with colonic J‐pouch reconstruction and concluded that a coloplasty pouch could be an alternative when colonic J‐pouch reconstruction was technically difficult (fat or a contracted pelvis making it difficult to reach the anus to create a large pouch).^22^ In an RCT by Ho et al comparing coloplasty pouches and colonic J‐pouch reconstruction, there were no significant differences in defecation or reservoir functions, but the anastomotic leakage rate was significantly higher with coloplasty pouches (16% vs 0%).^23^ The anastomotic leakages were all in the anterior wall of the anastomosis, which is directly beneath the coloplasty, and were caused by ischemia accompanying the longitudinal incision made in the intestine to create the coloplasty. They recommended leaving ≥4 cm between the colonic stump and the longitudinal incision, as well as creating a temporary stoma.^23^

As with coloplasty, side‐to‐end anastomosis was also confirmed around this time as an alternative to colonic J‐pouch reconstruction. Jiang et al conducted an RCT comparing 5‐cm colonic J‐pouch reconstruction and blind 5‐cm side‐to‐end anastomosis, reporting no significant differences in postoperative complications or long‐term defecation functions.^24^ Recently, Siddiqui et al conducted a meta‐analysis of four RCTs comparing colonic J‐pouch reconstruction and side‐to‐end anastomosis. They reported no significant differences in operation times, amount of blood loss, complications, hospital stays, reservoir functions, or defecation functions.^25^ Doeksen et al reported that, while postoperative defecation functions were slightly better with colonic J‐pouch reconstruction, the difference was not significant, and concluded that side‐to‐end anastomosis could be a technically simpler alternative to colonic J‐pouch reconstruction.^26^

Based on the evidences of the above, colonic J‐pouch and side‐to‐end anastomosis or coloplasty pouch lead to a better functional outcome than straight reconstruction after surgery for low rectal cancer. In addition, coloplasty pouch and side‐to‐end anastomosis had similar functional outcomes to the colonic J‐pouch.

#### 2.4.3 Intersphincteric resection (ISR)

Although often seen as a cutting‐edge procedure, the concept of partially resecting the sphincter and preserving the anus has a long history. The pull‐through procedure reported by Bacon in 1945 separated the internal and external sphincters and preserved the latter. Intersphincteric resection (ISR) was reported for the first time by Schiessel et al of Austria, who showed that it was possible to preserve the anus even in rectal cancers close to the anus.^27^ However, 2 years before this, a study from Japan by Kusunoki et al had already described four types of internal anal sphincter (IAS) resection (high partial resection, high circumferential resection, low partial resection, total internal sphincter resection) based on the tumor site, and performed physiological assessments of defecation functions using manometry.^28^ With colonic J‐pouch reconstruction performed in all cases, low partial/total sphincter resections tended to be inferior to high partial/circumferential resections in terms of defecation frequency and soiling mainly at night, though continence was maintained with all four procedures. In this important report, Kusunoki et al showed that anal static pressure was maintained in proportion to the degree to which the internal sphincter was preserved, thus demonstrating the significance of retaining as much of the internal sphincter as possible; they further described how colonic J‐pouches compensate for defecation functions. Yamada et al also conducted physiological assessments using manometry by dividing ISR into total, subtotal, and partial ISR.^29^ They reported that continence was maintained in 97% of cases, and that all these procedures could replace abdominal‐perineal rectal amputation. However, because functional decline was significantly greater with total ISR compared to the other procedures, careful preoperative evaluations of anal sphincter functions should be used for patient selection.

Tilney et al conducted a systematic review of the functional and oncological prognoses of ISR for lower rectal cancer. The total operative mortality rate was 1.6%, anastomotic leakage rate 10.5%, local recurrence rate 9.5%, and 5‐year survival rate 81.5%, indicating good short‐ and long‐term prognoses. Conversely, many cases exhibited reduced anal static pressure and frequent fecal urgency, though defecation functions and quality of life (QOL) were possibly improved by colonic J‐pouch reconstruction.^30^ A recent systematic review by Martin et al found that the complete resection rate of tumor was 97%, operative mortality rate was 0.8%, cumulative complication rate 25.8%, local recurrence rate 6.7%, 5‐year survival rate 86.3%, and 5‐year disease‐free survival rate 78.6%. Postoperative functions were good, with a mean 2.7 defecations per day. They concluded that although ISR for rectal cancer is oncologically feasible, it should be noted that it is associated with various forms of impaired defecation function.^31^

#### 2.4.4 Transanal local resection

The classic transanal resection for rectal cancer initially used a perineal approach, but this only resected the mucosal side of the tumor and did not remove the entire rectum including lymphoid tissue.

Kraske's master, Volkmann, described transanal resection as a procedure for localized rectal tumors with well‐defined borders.^32^ In the 1970s, Parks et al reported transanal local resection for villous tumors of the anal canal. In this technique, a surgical thread was placed around the anus and a retractor fixed in the anal canal to secure a visual field. Physiological saline solution containing adrenaline was injected submucosally, followed by transanal resection and suturing of the villous tumor.^33^  Transanal local resection as an excision biopsy method is currently the treatment for early stage rectal cancer. After performing pathological assessments of resected specimens, and considered a second resection to be necessary after evaluating the risk of lymph node metastasis.

"Guidelines 2019 for the Treatment of Colorectal Cancer" in Japan describes transanal local resection as the procedure for cTis and cT1 cancer (mild invasion) on the anal side of the peritoneal reflection, and recommends that, in principle, all layers should be resected.^34^ In addition to the conventional method of tumor resection under direct vision, Buess et al in 1984 developed transanal endoscopic microsurgery (TEM) as a method of making deep manipulations easier, and reported using TEM for rectal tumors.^35^ Later, Tsai et al reported the outcomes of 259 TEM cases, concluding that, as TEM techniques were safe and effective, they should be performed for benign diseases and cT1 rectal cancer.^36^ However, the use of TEM was not widespread due to the expensive and complex specialized instruments and forceps required, as well as the complicated techniques involved.

Recently, Atallah et al reported transanal minimally invasive surgery (TAMIS), in which a single port is made in the anal canal and local resection is performed with laparoscopic surgical instruments.^37^ Compared to TEM, TAMIS is expected to be more widely used because it does not require expensive specialized instruments and can be performed by surgeons with experience in regular laparoscopic surgeries.

## 3 ONCOLOGICAL SURGERY FOR RECTAL CANCER

Advancements in surgical procedures for rectal cancer since the Miles operation in 1908 have focused not only on preserving function but also on oncological aspects. Clinical research on lymph node metastasis and resected stumps has led to the development of total mesorectal excision (TME), which is currently the standard procedure for advanced rectal cancer. Here, we outline the history of this process.

### 3.1 Inferior mesenteric artery (IMA) ligation

The Miles operation involves ligation of the lower end of the left colic artery branch.^3^ In the 1930s, Dukes et al showed that lymphatic flow around rectal cancer travels from the IMA along the aorta, and suggested ligating the IMA at a high position.^38^ Later, Goligher's anatomical research showed that the mean distance from the IMA root to the left colic artery branch was 4 cm, and that there were approximately 10 lymph nodes in this area.^39^ The frequency of lymph node metastasis in this area has been reported as 11%‐22%, suggesting the importance of lymph node dissection with high IMA ligation.

In 1952, Grinnell et al performed lymph node dissections with high IMA ligation for rectal cancer,^40^ but ultimately concluded that this did not improve prognosis.^41^ In the 1980s, Pezim and Nicholls conducted a large study of 1370 cases of rectal cancer. In all Dukes stages, high IMA ligation was not found to be useful for improving the survival rate.^42^ A later report from the same institution also found that the level of IMA ligation did not contribute to improving prognosis after rectal cancer surgery.^43^ Similarly, the latest meta‐analysis (four RCTs, 20 cohort studies) did not find that high IMA ligation was useful for improving the survival rate.^44^ The reason for the lack of improvement in prognosis with high IMA ligation is that there are multiple other rectal lymph drainage routes in addition to the route proximal to the IMA, including the internal iliac and inguinal routes. In Europe and the USA, cases with multiple lymph node metastases at the IMA root are often considered to already have systemic disease.

### 3.2 Distal resection margin

After Miles first reported abdominal‐perineal rectal amputation for rectal cancer, Mayo recommended this as the standard procedure for all rectal cancers.^45^ In 1948, Dixon reported an anterior resection performed from the abdominal side with anastomosis of two layers. Called the Mayo Clinic operation, this was shown not only to be safer that abdominal‐perineal rectal amputation, but to have a good 5‐year survival rate (67.7%).^8^ In anterior resection, the distal resection margin is closely related to postoperative local recurrence. In 1948, Best et al proposed 3.5 mm as the first oncologically safe distal resection margin.^46^ In 1951, Goligher et al examined 1500 resected rectal cancer specimens, observing distal submucosal spread (mural spread) in 6.5%. The tumor's inferior margin extended by ≥2 cm in less than 2% of the specimens.^47^ In the 1980s and 1990s, based on histopathological research, an optimal distance of 2 cm was proposed for the anal side resection margin in rectal cancer. In a histological study of 334 cases of rectal cancer by Pollett et al, no significant differences in the survival rates or local recurrence rates were observed between 2‐cm and 5‐cm resection margins, providing evidence for an optimal margin of 2 cm.^48^ Similar studies from the 2000s found that, in lower rectal cancer, anal side progression exceeding 2 cm in the rectal wall and mesorectum was rare. It was thus recommended that a distal resection margin of this size be used. Further, an optimal anal side resection margin of 1 cm has been reported in cases of rectal cancer that received preoperative chemoradiotherapy (CRT).^49^

### 3.3 Circumferential resection margin and total mesorectal excision

Debating the optimal distal resection margin is meaningless if the circumferential resection margin (CRM) is positive. In 1982, Heald et al of Britain reported a groundbreaking procedure that they called total mesorectal excision (TME), prioritizing the CRM. In TME, the entire mesorectum contained in the visceral fascia is resected to the level of the levator ani.^50^ A 5‐year local recurrence rate of 5% was reported with TME for Dukes stages B and C rectal cancer without chemotherapy or radiation therapy; this was much better than the recurrence rates of 20%‐30% with conventional procedures.^51^ In addition, Havenga et al examined surgical outcomes of 1411 rectal cancer patients at five institutions, reporting local recurrence rates of 4%‐9% after introducing TME—a marked improvement over the 32%‐35% observed with conventional surgeries—as well as demonstrating a 30% additional effect on survival.^52^

For upper rectal cancer, TME extends the avascular wall on the anal side and increases the risk of anastomotic leakage. Therefore, the use of TME should be reserved for lower rectal cancers. Several reports on histopathological analyses of upper rectal cancer indicate that selective resection of the mesorectum 4‐5 cm on the anal side of the inferior margin of the tumor is as good as TME with respect to oncological prognosis.^53^, ^54^ This type of selective mesorectal resection is called selective TME or tumor‐specific TME (TSME). Japanese guidelines on colorectal cancer for 2019 recommend TME for the resection of rectal cancer. For upper rectal cancer, the recommended procedure is TSME so as to partially resect the mesorectum based on tumor location.

Recent CRM studies have reported significantly worse disease‐free survival when the CRM is <1 mm compared to cases of ≥1 mm.^55^ Similarly, there have been reports that CRM should be ≥2 mm to suppress local recurrence in preoperative CRT‐naive patients,^56^ and that CRM should be ≥1 mm in patients who have undergone preoperative CRT.^57^

Transanal minimally invasive surgery (TAMIS) was first reported in 2010 as an alternative to TEM for transanal polyp resection,^37^ but has evolved into a technique for performing TME in reverse (down‐to‐up) from the anal side. In 2013, de Lacy et al proposed this procedure as “down‐to‐up TME” and reported on short‐term results regarding postoperative complications in 20 cases.^58^ In a similar report from 2014, Atallah et al proposed TAMIS‐TME and concluded that the procedure was useful in obese (BMI > 30 kg/m^2^) patients with lower rectal cancer.^59^ Currently, TME performed transanally is called transanal total mesorectal excision (taTME).

In 2016, a report by the International Registry stated that the CRM‐positive rate was 2.4% among 720 patients who underwent taTME for rectal cancer.^60^ In recent years, several institutions have reported low CRM‐positive rates of 3.8%‐8% with taTME.^61^, ^62^ In a meta‐analysis, Jiang et al reported that taTME had a significantly higher radical resection rate than laparoscopic TME and highlighted the need for RCTs.^63^ In contrast, in a recent paper published in 2020 the data on all patients who underwent taTME were recorded and compared with those from national cohorts in the Norwegian Colorectal Cancer Registry and the Norwegian Registry for Gastrointestinal Surgery.^64^ As per the results, the rate of local recurrence, anastomotic leakage, and stoma construction were 7.6%, 8.4%, and 35.7%, respectively, which were significantly poorer results compared with previous national data for rectal cancer. Therefore, they concluded that even very experienced colorectal surgeons who performed taTME procedures had unsuccessful outcomes.^64^ In the near future, we need further prospective data to introduce taTME for rectal cancer.

### 3.4 Surgical therapies (lateral lymph node dissection) and multidisciplinary therapies (preoperative CRT) to suppress local recurrence of rectal cancer

#### 3.4.1 Lateral lymph node dissection

In Japan, lateral lymph node dissection to prevent local recurrence and improve prognosis has been widely performed since the 1970s. In the 1990s, the frequency of lateral lymph node metastasis in lower rectal cancer in Japan was reported to be 16%‐23%.^34^ The rate of local recurrence with lateral lymph node metastasis compared to mesorectal lymph node metastasis, as well as the belief that local control would be difficult with TME alone, provided the basis for performing extensive dissections. However, patients who underwent lateral dissection often experienced impaired urinary and, in male patients, sexual functions, and the 5‐year survival rate of these patients was approximately 30%‐40%. Therefore, lateral dissection has not been accepted in Europe or the USA.^65^

Ueno et al reported that cases of positive lateral lymph node metastasis were associated with tumor “budding” of the invasive front and vascular invasion, as well as a poor prognosis determined by the presence or absence of distant metastases.^66^ Further, Matsumoto et al showed that the prognosis was very poor when micrometastases were found in the dissected perineural tissue of patients who underwent autonomic nerve‐preserving lateral dissection, demonstrating the limitations of such dissection.^67^ Contrarily, an analysis of the propensity scores of pT3/T4 lower rectal cancer cases from 1995 to 2004 in the national registry of the Japanese Society for Cancer of the Colon and Rectum showed that the 5‐year overall survival rate of lateral dissection cases was better than that of non‐dissection cases (68.9% vs 62.0%).^68^ Furthermore, the JCOG0212 trial clarified the clinical significance of prophylactic lateral lymph node dissection in lower rectal cancer.^69^ As a result, non‐inferiority of TME alone compared to TME + lateral lymph node dissection was not demonstrated. Relapse free survival was 73.4% in the TME + lateral lymph node dissection group and 73.3% in the TME alone group (Hazard ratio (HR): 1.07, 95% CI: 0.84‐1.36, *P* = .055).^69^ In addition, the local recurrence rate was significantly lower in the TME + lateral lymph node dissection group, with a particularly marked decrease in the recurrence rate in the lateral region.^69^ Based on these results, Japanese guidelines for colorectal therapy recommended lateral lymph node dissection for rectal cancer when the inferior margin of the tumor is on the anal side of the peritoneal reflection and the wall depth is cT3 or greater.^34^

#### 3.4.2 Preoperative chemoradiotherapy

In Europe and the USA, preoperative CRT has become the standard treatment for controlling local recurrence after rectal cancer surgery. As this article is about surgical therapies for rectal cancer, what follows is a brief description of the clinical trials that support the use of CRT. (1) Short‐term preoperative irradiation (5 Gy × 5 days) of rectal cancer significantly reduces postoperative local recurrence. (2) Preoperative fractionated irradiation (50.4 Gy) of rectal cancer significantly reduces postoperative local recurrence compared to postoperative irradiation. (3) Preoperative fractionated irradiation (45 Gy) or combination with preoperative chemotherapy (5‐fluorouracil base) for rectal cancer significantly reduces local recurrence.^70^ This historically significant evidence suggests that while preoperative CRT has no added effect on survival, it significantly reduces the local recurrence rate of rectal cancer, which has made it the standard therapy worldwide.

#### 3.4.3 Watch and Wait after clinical complete response to chemoradiation

In 2004, Habr‐Gama et al of Brazil reported on advanced lower rectal cancer patients who underwent CRT with a clinical complete response (cCR) and were thus monitored without undergoing surgery.^71^ The results were interesting, with a local tumor regrowth rate of 2.8%. As salvage surgery remained an option for all patients, this extreme form of organ‐sparing therapy marked the appearance of the "watch and wait" strategy.^71^ Later results on the watch‐and‐wait strategy in rectal cancer were reported in a 2018 international multicenter registry study. A total of 880 patients were included from 47 centers across 15 countries, 87% of which exhibited cCR. Two‐year cumulative rates of local regrowth were noted in 25.2%. Eighty‐eight per cent of all local regrowth was diagnosed in the first 2 years, and 97% of local regrowth was located in the bowel wall. Five‐year overall survival was 85% with 5‐year disease‐free survival of 94%, which confirmed the reproducibility of the approach.^72^ In addition, the OnCore Project, published in 2016, was a propensity score‐matched cohort analysis study to compare watch and wait vs surgical resection.^73^ In the matched analyses (109 patients in each treatment group), no differences were noted in the 3‐year non‐regrowth disease‐free survival (88% with watch and wait vs 78% with surgical resection) and 3‐year disease‐free survival (96% vs 87%, respectively) rates.^73^ Watch‐and‐wait strategy is promising with regard to organ preservation in rectal cancer, and interest is growing fast. However, there is currently no level I evidence to support a watch‐and‐wait approach as standard in patients achieving cCR after nCRT for rectal adenocarcinoma.

#### 3.4.4 Local excision followed by chemoradiation for early rectal cancer

Endoscopic polypectomy or transanal local excision for favorable T1 tumors is the most accepted form of organ preservation and is considered the preferred treatment in Japanese guidelines.^34^ When at histology of the resection specimen the tumor has clear margins, is well/moderately differentiated, has no lymphatic or vascular invasion, and has only superficial invasion of submucosa (sm1‐2), the risk of lymph node metastases and local recurrence is below 5%. In contrast, in the presence of one or more adverse risk factors and when the tumor is larger than 3‐4 cm, this risk increases to 20%‐30%, and complete radical rectal surgery (TME) is recommended.^74^, ^75^ In patients who have a high operative risk or who refuse surgery, two alternatives can be considered: careful follow‐up with the option of salvage surgery when the residual disease appears, or adjuvant chemoradiation. A meta‐analysis reported that the rate of local recurrence was 5% in patients who received radiotherapy or CRT after local excision, which was similar to that (4%) in those who underwent total mesorectal excision (TME).^76^ In addition, recent systemic review revealed that local excision followed by adjuvant therapy can achieve acceptable long‐term outcomes in high‐risk pT1 rectal cancers (pooled local recurrence was 5.8% for pT1).^77^

#### 3.4.5 Chemoradiation followed by full thickness local excision

Local excision is considered a valid treatment option for very early tumors (pT1) without lymphatic spread. More advanced tumors have a higher risk of recurrence after a local excision compared with TME because of occult lymph node metastasis and intraluminal recurrences. The absence of viable tumor after neoadjuvant CRT led to a growing interest in alternative strategies for treating rectal cancer. Organ‐preserving treatment options aim for improving QOL with similar oncological outcome. Several retrospective studies describe the effect of local excision in patients who respond well to CRT for clinical (cT2‐3cN0‐1) rectal cancer, and it may be an equivalent to TME in selected patients with rectal cancer as far as long‐term oncological outcome is concerned.^78^ Recently, in the GRECCAR 2 study, patients with a good or complete response were randomized after CRT (cT2‐3N0 at primary staging) for local excision or TME.^79^ As the results indicate, the patients provided no evidence of difference in 5‐year oncological outcomes including local recurrence, overall survival, disease‐free survival, and cancer‐specific survival between local excision and TME, and concluded that local excision can be proposed in selected patients having a small cT2‐3 low rectal cancer with a good clinical response after CRT.

#### 3.4.6 Total neoadjuvant therapy

Although both TME with lateral lymph node dissection and CRT with TME can control to reduce local recurrence, no impact for improving survival has been found. Trials evaluating adjuvant chemotherapy for rectal cancer had disadvantages such as poor compliance rates and incompatible survival results.^80^ Therefore, shifting systemic therapy to the neoadjuvant setting has the promise to improve compliance rates, reduce toxicities, and decrease distant relapse rates. With the purpose of improving patient survival, delivery of chemotherapy before surgery had been proposed to treat occult micrometastases early and increase treatment compliance.^81^ Multiple trials evaluating various modes of incorporating both chemotherapy and CRT in the neoadjuvant setting, referred to as “total neoadjuvant therapy (TNT),” have reported optimistic results, including higher pathological complete response, better disease‐free survival, and overall survival.^82^ According to the above evidences, NCCN guidelines categorize TNT as a viable treatment strategy for rectal cancer. In addition to improving survival, TNT has the potential to increase the population of patients with rectal cancer who are eligible for the watch‐and‐wait strategy.

## 4 ADVANCES IN MINIMALLY INVASIVE SURGERY

### 4.1 Laparoscopic surgery

Laparoscopic surgery was first performed on the appendix and gallbladder in the 1980s, and in the 1990s was introduced for colorectal surgery. In 1991, Jacobs et al reported the world's first laparoscopic surgery case for colorectal cancer.^83^ Many retrospective analyses have shown that laparoscopic surgery is less invasive than laparotomy and is not inferior in terms of the long‐term prognosis. The COREAN trial^84^ and COLORII trial^85^ reported no differences in survival rates between laparotomy and laparoscopic surgery for rectal cancer. In contrast, neither the ACOSOG Z6051 trial^86^ nor the ALaCaRT trial^87^ showed equivalent oncological resection success rates with laparoscopic surgery. A meta‐analysis of 14 RCTs failed to show that the oncological resection success rate of laparoscopic surgery was the same as or better than that of laparotomy for rectal cancer, and suggested a higher risk of incomplete TME and positive CRM with the former.^88^ The validity of the oncological prognosis of laparoscopic surgery for rectal cancer thus still needs to be verified.

### 4.2 Abdominal robot‐assisted surgery

In the 1980s, the US Military began developing the da Vinci^®^ surgical robot for telemedicine during combat. Its development was handed over to a private company and completed in 1999. In 2000, the US Food and Drug Administration approved the da Vinci^®^ as a surgical robot.^89^ Robot‐assisted surgery with the da Vinci^®^ for colorectal cancer was first reported in Japan by Hashizume et al in 2002.^90^ In 2003, the da Vinci^®^ was used for rectal cancer for the first time, to perform anterior resection with abdominal‐perineal rectal amputation.^91^ Kim et al reported that robotic surgery can be used to perform delicate operations on blood vessels and nerves, and helps to significantly preserve urinary and sexual functions after rectal cancer surgery.^92^ Yamaguchi et al examined lateral lymph node dissection, showing that robot‐assisted surgery reduced the amount of bleeding and incidence of urinary retention and contributed to reducing local recurrence compared to laparotomy.^93^ Conversely, an RCT (ROLARR trial) comparing robotic and laparoscopic surgery for rectal cancer did not find robotic surgery to be superior in terms of the rate of conversion to laparotomy.^94^ A meta‐analysis comparing robot‐assisted and laparoscopic surgery for rectal cancer showed a lower rate of conversion to laparotomy and a higher point of technical limitation with robot‐assisted surgery, but no difference in short‐term postoperative outcomes or long‐term prognosis. An RCT comparing robot‐assisted and laparoscopic surgery is in progress. Since 2018, robot‐assisted surgery for rectal cancer has been covered by insurance in Japan, and the number of institutions specializing in these procedures is expected to increase; however, the expensive surgical equipment and other issues involving medical economics remain problematic.

## 5 CONCLUSION

This has been a review of the history of surgical therapies for lower rectal cancer spanning more than 100 years. This article covered changes in the surgical approaches to rectal cancer and changes in surgical styles from the standpoints of oncology, anal function, and defecation function, as well as the development of minimally invasive surgeries using laparoscopy and robotic assistance. All of this evidence was accumulated through our predecessors' devotion to their patients and unceasing dedication to the scientific work of clinical trials aimed at improving survival rates, increasing postoperative QOL, and preserving physiological functions.

In the future, we anticipate dramatic advancements in molecular biology, as well as in tailor‐made surgical therapies through the introduction of artificial intelligence in medicine. Decisions regarding surgical indications, resection range, and extent of lymph node dissection may move from the current TNM classifications to depend greatly on molecular biology. In addition, the fusion of artificial intelligence with medical robots and other new devices is expected to diversify surgical environments. However, advances in medicine, particularly in surgical science, for consulting with patients and confronting their diseases will always be built on wisdom gained through the accumulated efforts of our predecessors. While remaining ever‐cognizant of where our knowledge comes from, we await further developments in surgical therapies for rectal cancer.

## DISCLOSURE

Conflict of Interest: Authors declare no conflict of interests for this article.
